# Supplementary material for: Adoption of K-means clustering algorithm in smart city security analysis and mythical experience analysis of urban image
Source: PLoS One. 2025 Mar 10;20(3):e0319620. doi: 10.1371/journal.pone.0319620 (PMC11892831; doi:10.1371/journal.pone.0319620)
Supplement: Attachment — Submitted filename: Response_to_Reviewers_auresp_2.docx [file pone.0319620.s003.docx]

Dear Editor and Reviewers：

Thank you and the reviewers for your valuable comments on our manuscript *Adoption of K-Means Clustering Algorithm in Smart City Security: Analysis and Mythical Experience Analysis of Urban Image*. We have carefully read the reviewers’ comments and made corresponding modifications and improvements to the manuscript according to the feedback. We now submit the revised manuscript to you, along with the following contents:

1.	Response Letter: We have replied to each comment raised by the academic editor and reviewers, and have provided detailed explanations of the modifications made in the text. The response letter has been uploaded, with the file name “Response to Reviewers”.

2.	Revised Manuscript with Track Changes: We have uploaded a copy of the manuscript with track changes, so that the reviewers can clearly find all the modifications we have made to the article. The file name is “Revised Manuscript with Track Changes”.

3.	Revised Manuscript without Track Changes: In addition, we have also uploaded a copy of the manuscript without track changes. The file name is “Manuscript”.

We have further optimized and explained the experimental design according to the reviewers’ suggestions.

In addition, storing the laboratory experimental protocol on the protocols.io platform is not applicable.

Thank you again for your recognition of our research and for the careful review of the manuscript. We look forward to your further feedback.

**Comments to the Author**

1. If the authors have adequately addressed your comments raised in a previous round of review and you feel that this manuscript is now acceptable for publication, you may indicate that here to bypass the “Comments to the Author” section, enter your conflict of interest statement in the “Confidential to Editor” section, and submit your "Accept" recommendation.

Reviewer #2: All comments have been addressed

Reviewer #4: All comments have been addressed

2. Is the manuscript technically sound, and do the data support the conclusions?

The manuscript must describe a technically sound piece of scientific research with data that supports the conclusions. Experiments must have been conducted rigorously, with appropriate controls, replication, and sample sizes. The conclusions must be drawn appropriately based on the data presented.

Reviewer #2: Yes

Reviewer #4: Yes

3. Has the statistical analysis been performed appropriately and rigorously?

Reviewer #2: Yes

Reviewer #4: Yes

4. Have the authors made all data underlying the findings in their manuscript fully available?

The [PLOS Data policy](http://track.editorialmanager.com/CL0/http:%2F%2Fwww.plosone.org%2Fstatic%2Fpolicies.action%23sharing/1/010f01943f0ddc76-a3b1eade-3a8c-4002-9e8f-dfe2594958ce-000000/CZV2w1P2GdjdkrDXcWeRFpZc51GsCz2y-g9iH6vyhVw=192) requires authors to make all data underlying the findings described in their manuscript fully available without restriction, with rare exception (please refer to the Data Availability Statement in the manuscript PDF file). The data should be provided as part of the manuscript or its supporting information, or deposited to a public repository. For example, in addition to summary statistics, the data points behind means, medians and variance measures should be available. If there are restrictions on publicly sharing data—e.g. participant privacy or use of data from a third party—those must be specified.

Reviewer #2: Yes

Reviewer #4: Yes

5. Is the manuscript presented in an intelligible fashion and written in standard English?

PLOS ONE does not copyedit accepted manuscripts, so the language in submitted articles must be clear, correct, and unambiguous. Any typographical or grammatical errors should be corrected at revision, so please note any specific errors here.

Reviewer #2: Yes

Reviewer #4: Yes

6. Review Comments to the Author

Please use the space provided to explain your answers to the questions above. You may also include additional comments for the author, including concerns about dual publication, research ethics, or publication ethics. (Please upload your review as an attachment if it exceeds 20,000 characters)

Reviewer #2: I checked the revised version. I noticed that you answered all the revisions correctly. Now it seems that this manuscript has reached an acceptable level in terms of quality.

Reviewer #4: Comments on the manuscript entitled
“Adoption of K-Means Clustering Algorithm in Smart City Security: Analysis and Mythical Experience Analysis of Urban Image”
The manuscript presents a well-structured and coherent proposal for a hybrid model, demonstrating an improvement in accuracy over traditional models such as logistic regression and Naive Bayes. The authors have adequately addressed the reviewers' queries, indicating a thorough understanding of the research domain. In my opinion, it can be published in the journal after addressing the following comments/suggestions.

Some Comments/ Recommendations
1. While the model's performance is superior to simpler models e.g. LR and NB, it does not exhibit a significant improvement over more complex models like SVM and Random Forest. It would be beneficial for the author(s) to explore and discuss the possible level of interpretability of their proposed model in relation to those of counterparts mentioned.

Reply: We appreciate the reviewer’s suggestions. While the C4.5 decision tree model based on KMC performs excellently when compared with simpler models such as LR and NB, the performance improvement is not significant when compared with more complex models like SVM and random forest. To better understand and apply this model, we will further analyze its interpretability in the discussion. Specifically, the tree structure of the C4.5 decision tree model is easy to understand and interpret, which gives it an advantage in transparency and interpretability of the decision-making process compared to models like SVM and random forest. We will further discuss this point and provide a comparison of interpretability with models such as SVM and random forest.
2. First, there is no explanation on how the data has been splitted to train/test sets. Then, given the relatively small number of observations, it's essential to assess whether the models, particularly the new one, suffer from overfitting or underfitting or not.
Reply: We agree with the reviewer’s concern about the division of the training set/test set. We will clarify the method of dataset division in the revision, adopting the common 10-fold cross-validation method to divide the data to ensure the stability and generalization ability of the model. In addition, due to the small number of observations, we have added an assessment of overfitting and underfitting in the results section.
Additional Remarks
• The formulae, specifically (1), (2), and (7), could be rewritten for clarity and consistency, potentially following the format of (8).

Reply: We appreciate the reviewer’s suggestions regarding the clarity and consistency of the formulas. In the revision, we will rewrite formulas (1), (2), and (7) according to the reviewer’s suggestions to ensure that they are expressed more clearly and in accordance with the principle of consistency. Specifically, we will refer to the format of formula (8) and make the layout of the formulas more intuitive, ensuring that each variable and operation in the formulas clearly and explicitly conveys its mathematical meaning.
• Line 315: Simplify the notation to "F1-F20" for better readability.

Reply: We agree with the reviewer’s suggestion to simplify the notation “F1-F20”. In the revision, we will directly use “F1-F20” to simplify the description, making it more concise and easier to understand. This will enhance the readability of the article and avoid redundant expressions.
• Eq (4): distance

Reply: Regarding the issue of distance calculation mentioned in formula (4), we have clarified and rewritten the formula to ensure its consistency with the model’s computation.

7. PLOS authors have the option to publish the peer review history of their article ([what does this mean?](https://track.editorialmanager.com/CL0/https:%2F%2Fjournals.plos.org%2Fplosone%2Fs%2Feditorial-and-peer-review-process%23loc-peer-review-history/1/010f01943f0ddc76-a3b1eade-3a8c-4002-9e8f-dfe2594958ce-000000/DTCLh5q1U9InbBCgHUPHR_CFxsHOWdU3qb9IlHJpTCw=192)). If published, this will include your full peer review and any attached files.


If you choose “no”, your identity will remain anonymous but your review may still be made public.


**Do you want your identity to be public for this peer review?** For information about this choice, including consent withdrawal, please see our [Privacy Policy](https://track.editorialmanager.com/CL0/https:%2F%2Fwww.plos.org%2Fprivacy-policy/1/010f01943f0ddc76-a3b1eade-3a8c-4002-9e8f-dfe2594958ce-000000/9UyMvbFEH3MDsCiRQGPZ7x9eCn8zeEeBq5AOYKBWmR8=192).

Reviewer #2: **Yes:**

Reviewer #4: **Yes:**
