# Supplementary material for: Adoption of K-means clustering algorithm in smart city security analysis and mythical experience analysis of urban image
Source: PLoS One. 2025 Mar 10;20(3):e0319620. doi: 10.1371/journal.pone.0319620 (PMC11892831; doi:10.1371/journal.pone.0319620)
Supplement: Attachment — Submitted filename: Response to Reviewers.docx [file pone.0319620.s002.docx]

Dear Editor and Reviewers：

We would like to express our sincere gratitude to you and the reviewers for your thoughtful evaluation of our manuscript and for the invaluable feedback provided. We highly appreciate each suggestion and have carefully revised the manuscript in accordance with the reviewers' and editors' comments. We are now submitting the revised manuscript for your further consideration.

To facilitate your review, we are providing the following documents:

1. **Response to Reviewers** – This document contains a point-by-point response to each of the reviewers' and editor’s comments, offering a detailed explanation of the modifications made.
2. **Revised Manuscript with Track Changes** – The manuscript with all revisions highlighted, showing the specific changes made, has been uploaded and labeled as “Revised Manuscript with Track Changes.”
3. **Clean Revised Manuscript** – A clean version of the manuscript for the convenience of the editor and reviewers, labeled as “Manuscript.”

Additionally, following a thorough analysis of your research methodology, we believe that the current methods are not fully suitable for storage on the protocols.io platform, for the following reasons:

1. **Lack of Specificity and Operability in Experimental Steps**: protocols.io is primarily designed for storing detailed experimental protocols and laboratory workflows that are reproducible. However, the methodology in this study involves the Delphi method, surveys, and stratified sampling—social science data collection methods that do not include specific experimental steps, making them difficult to standardize into a reproducible experimental workflow.
2. **Limited Reproducibility**: The Delphi method and the resident survey in this study are highly context-dependent, influenced by specific populations, timeframes, and environments. This makes it challenging to ensure full reproducibility in different settings. protocols.io is better suited for highly reproducible and controlled experimental methods, rather than social science data collection processes like the ones employed in this study.
3. **Insufficient Standardization of Steps**: While the K-Means and C4.5 decision tree algorithms used in this study have some degree of repeatability, these methods are more appropriate for sharing as data analysis techniques in research papers or on data analysis platforms. Detailed algorithm descriptions and parameter settings are better suited for publication rather than being stored as experimental steps on protocols.io.

Therefore, we conclude that protocols.io is not suitable for storing the methodology presented in this study. We are grateful for the opportunity to revise our manuscript and look forward to your further feedback.

**Journal requirements:**

When submitting your revision, we need you to address these additional requirements.

1. Please ensure that your manuscript meets PLOS ONE's style requirements, including those for file naming. The PLOS ONE style templates can be found at

<https://journals.plos.org/plosone/s/file?id=wjVg/PLOSOne_formatting_sample_main_body.pdf>

and <https://journals.plos.org/plosone/s/file?id=ba62/PLOSOne_formatting_sample_title_authors_affiliations.pdf>

Reply: We sincerely appreciate your valuable suggestions regarding our manuscript. We have carefully reviewed and adjusted the manuscript format to ensure compliance with the style guidelines of *PLOS ONE*, including proper file naming conventions. The overall structure and formatting of the manuscript have been revised in accordance with the template you provided.

2. We note that your Data Availability Statement is currently as follows: [All relevant data are within the manuscript and its Supporting Information files.]

Please confirm at this time whether or not your submission contains all raw data required to replicate the results of your study. Authors must share the “minimal data set” for their submission. PLOS defines the minimal data set to consist of the data required to replicate all study findings reported in the article, as well as related metadata and methods (<https://journals.plos.org/plosone/s/data-availability#loc-minimal-data-set-definition>).

Reply: We confirm that the current submission includes the minimal dataset supporting the research findings, the data points used for the figures, and the data points involved in the image analysis. All data have been uploaded as supporting information files. Should any further detailed information be required, we are happy to provide additional materials as needed.

3. PLOS requires an ORCID iD for the corresponding author in Editorial Manager on papers submitted after December 6th, 2016. Please ensure that you have an ORCID iD and that it is validated in Editorial Manager. To do this, go to ‘Update my Information’ (in the upper left-hand corner of the main menu), and click on the Fetch/Validate link next to the ORCID field. This will take you to the ORCID site and allow you to create a new iD or authenticate a pre-existing iD in Editorial Manager.

 Reply: We sincerely appreciate your suggestions regarding our manuscript. The corresponding author has verified their ORCID iD in Editorial Manager to ensure the uniqueness and traceability of the authorship.

**Reviewer #1**: The comments are as follows:

1) The manuscript compares the proposed model with Naive Bayes (NB) and Logistic Regression (LR) models. Why were more advanced machine learning models, such as Random Forests, Support Vector Machines (SVM), or Gradient Boosting Machines (GBM), not included in the comparison? How might the results differ if these more sophisticated models were considered?
Reply: We thank the reviewer for their insightful suggestions. In our study, we chose to compare the Naive Bayes (NB) and Logistic Regression (LR) models because these models are widely used both theoretically and practically, and they offer strong interpretability. However, we acknowledge that more complex models may yield different results. Therefore, we have also included models with higher complexity and potential for improved performance, such as Random Forest, Support Vector Machine (SVM), and Gradient Boosting Machine (GBM), for a comparative analysis of the results.
2) The study uses ROC curves as a performance metric, which can be misleading in cases of imbalanced datasets. How does the proposed model address potential class imbalance in the data, and why were other metrics, such as precision-recall curves or the F2 score, not considered?
Reply: We appreciate the reviewer’s observation. In the presence of class imbalance in the dataset, we acknowledge that the ROC curve may not fully reflect the model's performance. To address this, we have taken the class imbalance into consideration and performed preprocessing using oversampling techniques such as SMOTE. We also recognize that metrics such as the Precision-Recall Curve (PRC) and F2 score are more effective for evaluating the performance of models on imbalanced datasets. As a result, we have included the Precision-Recall Curve (PRC) as an additional evaluation metric.

3) The study uses data from 15 smart cities to validate the model. Is this sample size sufficient to generalize the findings to other smart cities globally, especially given the diverse nature of smart city implementations? How was the sample size determined, and what statistical methods were used to ensure it is representative?
Reply: We thank the reviewer for their valuable comments regarding the sample size. We acknowledge that, given the diversity of global smart cities, the initial selection of 15 smart cities for validation may not fully represent the entire spectrum of smart cities. The sample size was primarily determined based on the city rankings in the *China Smart City Development Level Assessment Report*, with the aim of selecting representative cities with high levels of development. To enhance the generalizability of our findings, we have subsequently increased the sample size to 38 cities.

4) Introduction is not focused and literature can be reorganised to strengthen literature review following contributions and discuss few relevant works i.e.,
a) Deep learning models for intelligent healthcare: implementation and challenges
b) Unsupervised pre-trained filter learning approach for efficient convolution neural network
c) CSFL: A novel unsupervised convolution neural network approach for visual pattern classification
d) Optimization of CNN through novel training strategy for visual classification problems
e) Face recognition: A novel un-supervised convolutional neural network method
Reply: We sincerely thank the reviewer for their valuable comments. In response to your suggestions, we will reorganize the literature review section and incorporate the relevant studies you mentioned to strengthen the theoretical framework and background analysis. We will pay particular attention to the latest advancements in deep learning, such as those in smart healthcare and Convolutional Neural Network (CNN) optimization, as well as their potential connections to risk assessment in smart cities.

5) The results indicate that cities with the same attributes exhibit minor differences in risk levels, while cities with different attributes show significant variations. Can the authors provide a more detailed explanation of what these differences in attributes entail and how they specifically contribute to variations in risk levels?

Reply: We appreciate the reviewer’s insightful question. In our study, the differences in risk levels between cities are primarily attributed to variations in their social, economic, and technological characteristics, such as the level of information infrastructure, residents' digital literacy, and the economic development level. These factors directly influence the challenges and risks associated with information security management in smart cities. In the revised manuscript, we will provide a more detailed explanation of these attributes and explore how they impact the risk levels of smart cities through different mechanisms.

**Reviewer #2:** I reviewed the manuscript titled 'Adoption of K-Means Clustering Algorithm in Smart City Security: Analysis and Mythical Experience Analysis of Urban Image'. After a careful examination, I found that the manuscript is complete and well-written. The methodology used in the study is highly accurate and valuable. The research questions have been addressed effectively. The content presented in the manuscript has been meticulously collected. Based on these observations, it appears that the study can be accepted in its current form."

**Reviewer #3**: This paper evaluates information security in smart cities (SC) through a combined K-Means clustering and C4.5 decision tree model, examining not only security assessments but also residents' mythological experiences in these cities. The following review highlights the paper’s strengths and areas for improvement:
Strengths

Innovative Methodology: The use of a combined K-Means clustering and C4.5 decision tree for information security assessment in smart cities is a novel approach that could offer new insights into security evaluations.
Strong Results and Model Comparison: The paper reports high accuracy and an AUC greater than 0.9 for the combined model. Comparisons with other methods, such as Naïve Bayes (NB) and Logistic Regression (LR), further support the effectiveness of the combined model.
Cultural and Mythological Analysis: Integrating an analysis of cultural and mythological experiences with urban security is an original approach that adds a unique dimension, highlighting the potential relationship between culture and security in urban spaces.

Weaknesses

Lack of Clear Link Between Security and Culture: While the paper attempts to connect security and cultural experiences in smart cities, this relationship remains somewhat unclear. More detailed explanations on how information security is related to mythological experiences would add clarity.
Insufficient Details in Methodology: The methodology section is somewhat brief. For instance, further explanation on the Delphi method, the selection of experts, and other assessment steps would enhance the reader's understanding and confidence in the findings.
Need for More Comprehensive Data: The study focuses on only 15 cities, which may be insufficient for drawing broader conclusions on urban information security and cultural experiences. Expanding security assessment indicators and including additional variables would strengthen the model.

Suggestions for Improvement

Expand Methodology Details: Providing further details on data collection, standardization, and the rationale for selecting the combined K-Means and C4.5 model would give readers a clearer understanding of the model's effectiveness.

Reply: Thank you for your suggestion. I will provide additional details regarding the data collection process, the standardization procedures, and the rationale for selecting the combination of K-Means and C4.5 models. This will help to more clearly demonstrate the effectiveness of the models and enhance the reader's understanding.
Increase Sample Size and Data Variety: Expanding the study to cover more cities and examine additional indicators would enhance the generalizability of the results.

Reply: Thank you for your suggestion. I plan to expand the scope of the study to include more cities and examine additional indicators. This will not only increase the sample size but also enhance the generalizability of the results, making the findings more applicable across different geographic regions and contexts.

Clarify the Link Between Security and Culture: Clearer explanations on how information security impacts cultural and spiritual experiences of city residents could enhance the paper’s value and originality.
Reply: Thank you for your reminder. I will further elaborate on how information security impacts the cultural and psychological experiences of urban residents. This addition will help highlight the originality of the paper and more clearly demonstrate the importance of the relationship between security and culture in the context of the study.

While revising your submission, please upload your figure files to the Preflight Analysis and Conversion Engine (PACE) digital diagnostic tool, <https://pacev2.apexcovantage.com/>. PACE helps ensure that figures meet PLOS requirements. To use PACE, you must first register as a user. Registration is free. Then, login and navigate to the UPLOAD tab, where you will find detailed instructions on how to use the tool. If you encounter any issues or have any questions when using PACE, please email PLOS at <a href="mailto:figures@plos.org">figures@plos.org. Please note that Supporting Information files do not need this step.

Reply: Thank you for your guidance. I will upload the figure and table files to the PACE tool as required and ensure that they comply with PLOS's standards. For the specific steps involved in using PACE, I will follow the provided instructions. If any issues arise, I will contact PLOS for assistance. Additionally, I understand that supporting information files do not require this step. Should it be necessary to delete personal registration information, I will follow the instructions and reach out to the publishing office.
